# Dyadic Risk and Protective Factors of Caregiver Burden Among Partners of Patients With Advanced Cancer: A Network Approach

**DOI:** 10.1002/pon.70403

**Published:** 2026-02-05

**Authors:** Melanie P. J. Schellekens, Dounya Schoormans, Moyke Versluis, Meeke Hoedjes, Natasja J. H. Raijmakers, Marije L. van der Lee, Floortje Mols

**Affiliations:** ^1^ Department of Medical and Clinical Psychology Tilburg University Center of Research in Psychological Disorders and Somatic Diseases (CoRPS) Tilburg the Netherlands; ^2^ Scientific Research Department Helen Dowling Institute Expert Centre for Psycho‐Oncology Utrecht the Netherlands; ^3^ Department of Research & Development Netherlands Comprehensive Cancer Organisation Utrecht the Netherlands

## Abstract

**Objective:**

To examine how dyadic patient‐ and partner‐related risk and protective factors are interconnected with caregiver burden among partners of patients with advanced cancer using a network approach.

**Methods:**

We conducted network and shortest‐path analyses using cross‐sectional baseline data from the eQuiPe study, including 564 patient–partner caregiver couples. The network included patient‐ and partner‐reported physical, emotional, and sleep problems, social and partner support, continuity of care, and caregiver burden.

**Results:**

Shortest‐path analysis identified patient‐perceived continuity of care as the only patient‐related protective factor directly connected to lower caregiver burden. Patients' physical problems were indirectly linked to caregiver burden via emotional problems of both patients and partners.

**Conclusions:**

Continuity of care and the interdependence between patient and partner emotional problems appeared to be important dyadic protective and risk factors of partners' caregiver burden. Improving continuity of palliative care and offering dyadic interventions targeting emotional functioning of both partners may help reduce caregiver burden. To further improve our understanding of caregiver burden and its dyadic factors, future studies should apply intensive longitudinal designs to explore how these components interact over time.

## 1 Background

Across the cancer trajectory, partner caregivers play a central role in supporting patients with advanced cancer, often with substantial psychosocial consequences for their own well‐being [1]. With increasing healthcare shortages, the responsibility of caring for patients with advanced cancer is increasingly placed on partners [2]. Partner caregivers carry multiple responsibilities over a long period of time, including the provision of emotional support, assistance with activities of daily living, and medical procedures at home [3]. Often, partner caregivers are not adequately prepared or equipped for these tasks and many experience significant caregiver burden [4]. Within the eQuiPe cohort, previous analyses have shown that approximately one‐third of caregivers of patients with advanced cancer experience high caregiver burden [4], which is associated with anxiety, depression, feelings of social isolation, and low quality of life [4, 5].

Caregiver burden is a multi‐dimensional construct encompassing emotional, physical, social, and role‐related strain [6]. While emotional distress is a central component, caregiver burden also reflects disruptions in daily functioning, social relationships, and perceived responsibility. In the present study, we primarily focus on emotional and psychosocial dimensions of caregiver burden, given the available data and the strong evidence linking emotional distress to caregiver burden in advanced cancer.

High levels of caregiver burden do not only affect caregivers themselves, but may also compromise the quality of care provided to patients [7]. Overburdened caregivers may have reduced capacity to monitor symptoms, communicate effectively with healthcare professionals, or support treatment adherence, which may ultimately affect patients' quality of life and care outcomes [7]. This underscores the clinical importance of identifying modifiable risk and protective factors of caregiver burden.

To adequately understand caregiver burden in advanced cancer, it is essential to adopt a dyadic perspective that acknowledges the interdependence between patients and their partners. This requires insight into risk and protective factors in both the partner and the patient, as partners in long‐term relationships are theorized to be interdependent, mutually affecting each other [8]. A meta‐analysis of 35 studies (2468 couples) coping with cancer revealed a moderate association (*r* = 0.29) between patients' and spouses' psychological distress [9]. Similarly, in couples facing advanced cancer, the coping of one partner affects the other partner's emotional functioning and ability to cope, and vice versa [10]. Together, these findings show that couples coping with cancer function as an interdependent emotional system, underscoring the need for a dyadic approach to studying caregiver burden in advanced cancer.

The transactional theory of stress and coping offers a well‐established framework to understand how partners adapt to advanced cancer and their caregiving role [11]. The theory posits that a diagnosis of advanced cancer can lead to caregiver burden among partners, due to the appraisal of stressors, resources and coping with advanced cancer and the caregiver role. In line with the stress and coping theory, several studies have successfully identified factors that put partner caregivers at risk or protect them from the development and persistence of caregiver burden [5]. Specifically, the theory posits that during primary appraisal, the partner judges whether the situation poses a threat. Numerous studies have found that the primary threat, such as the level of patients' physical and emotional problems, contribute to caregiver burden [12]. In addition, the secondary appraisal assesses one's ability to cope with the perceived threat and analyze which resources are available and what coping mechanisms would be most effective.

Emotional problems will limit the caregiver's ability to cope with the stressors of advanced cancer. Studies confirmed emotional problems are associated with heightened caregiver burden [13]. A valuable resource that has been highlighted by multiple studies as significant for both patients and partner caregivers is continuity of care (i.e., the extent to which the care of different healthcare professionals aligns with one another) [14, 15]. In addition, coping styles, such as turning to one's social network or partner for support, have been shown to serve as a protective factor of caregiver burden [16].

Caregiver burden in advanced cancer can be best understood as the result of a set of interrelated risk and protective factors operating within patient–partner dyads. While most studies examined these constructs as independent predictors of caregiver burden, it is more likely that these different risk and protective factors are interconnected among dyads. For example, patients who have more emotional problems are likely to report more sleep problems. In turn, when a partner caregiver's sleep is disturbed because of the patient's sleep problems, they are more likely to experience higher caregiver burden. Therefore, we propose to apply the network approach to gain a comprehensive understanding of how caregiver burden, and its dyadic risk and protective factors are interconnected. The network approach conceptualizes symptoms and its risk and protective factors, as mutually interacting and reinforcing components of a complex dynamic system [17]. Network modeling can be applied to cross‐sectional designs to study the co‐occurrence of certain concepts at the group level, offering insight into patterns of symptoms across individuals as well as dyads [18].

### 1.1 Aim

The objective of this study was to examine how dyadic patient‐ and partner‐related risk and protective factors are interconnected with caregiver burden among partner caregivers of patients with advanced cancer. Using network and shortest‐path analyses, we aim to identify which factors are directly and indirectly connected to caregiver burden in order to identify targets for supportive interventions.

## 2 Methods

We followed the guidelines for reporting network analysis of cross‐sectional data [19] and the STROBE reporting standards for observational research [20]. The study, data analysis plan, and code were preregistered online on the Open Science Framework prior to data analysis (https://osf.io/eqatu/).

### 2.1 Study Design and Participants

The eQuipe study is a prospective, longitudinal, observational study conducted in the Netherlands, designed to evaluate the quality of care and quality of life among patients with advanced or metastatic cancer receiving palliative care and their relatives [21].

Patient inclusion criteria were as follows: adults diagnosed with a solid metastasized (stage IV) tumor who were able to complete a Dutch questionnaire, comprehend the study purpose, and provide informed consent. All primary tumor types were eligible; however, to ensure comparable prognoses, patients with breast cancer were included only if their disease was metastasized to multiple organs, and patients with prostate cancer only if their disease was castrate resistant. Exclusion criteria comprise dementia or severe psychiatric illness.

Relatives of patients, as chosen by the patient, were also invited to participate.

For the present study, we only included patient and partner caregiver couples, defined as patients with advanced cancer and their romantic partners (e.g., spouses or long‐term partners), regardless of legal marital status or sexual orientation, who were identified by the patient as their primary informal caregiver. This focus on partner caregivers was chosen because romantic partners typically share daily life with the patient and are most strongly embedded in dyadic emotional and caregiving processes, aligning with the theoretical focus of the present study.

### 2.2 Ethical Considerations

The study was conducted in accordance with the Declaration of Helsinki. The study protocol was reviewed by the Medical Ethical Committee of the Netherlands Cancer Institute (NKI) (METC17.1491). The committee exempted this observational research from full ethical review in accordance with the Dutch Medical Research Involving Human Subjects Act (WMO). Data collection and analysis procedures adhered to the Dutch Personal Data Protection Act and the General Data Protection Regulation (GDPR). In all participating hospitals, local scientific committees—where applicable—were contacted for approval. Written informed consent was obtained from all participants.

### 2.3 Recruitment

Participants were recruited through 40 Dutch hospitals between 2017 and 2021.Healthcare professionals identified eligible patients, provided an information leaflet, and asked permission to share contact details with the research team. Patients could also self‐refer through the Dutch cancer information platform www.kanker.nl. A researcher then contacted interested patients and relatives by phone within a few days to explain the study and discuss participation.

### 2.4 Data Collection

After providing written informed consent, participants completed a baseline questionnaire, followed by 3‐monthly follow‐up questionnaires until death or study end (March 2020) as part of the longitudinal eQuiPe study. All questionnaires could be completed on paper or digitally via the PROFILES registry [22]. Questionnaire data were linked with clinical information (i.e., tumor type and stage, and treatment history) from the Netherlands Cancer Registry (NCR). Data for the present analyses were exclusively drawn from the baseline assessment and analyzed cross‐sectionally.

### 2.5 Variable Selection and Questionnaires

After several discussions among a subgroup of authors, we selected specific psychosocial and care‐related domains from the baseline data of the eQuiPe study that were considered theoretically and clinically relevant for inclusion in the network. To avoid problematic overlap between variables, we examined bivariate correlations and planned to average items with strong conceptual similarity and correlations of *r* ≥ 0.60 [19]. With bivariate correlations ranging between −0.322 and 0.506 this was not necessary. In total, 10 variables were included: caregiver burden (partner), risk factors, including physical problems (patient), sleep problems (patient), emotional problems (partner and patient), and protective factors, including continuity of patient's care (partner and patient), social support (partner), partner support (partner and patient). Each variable was assessed using a validated instrument. Table 1 provides an overview of the variables and questionnaires of partner caregivers and patients.

**TABLE 1:** Variables included in the network.

Variables and questionnaires	Range	Partner caregiver M (SD)	Patient M (SD)	*p*‐value
Caregiver burden (Zarit‐12)	0–48	10.00 (7.19)		NA
Risk factors				
Emotional problems (EORTC‐QLQ‐C30)	0–100	30.65 (21.66)	22.69 (21.29)	< 0.001
Physical problems (EORTC‐QLQ‐C30)	0–100		29.32 (22.39)	NA
Sleep problems (EORTC‐QLQ‐C30)	0–100		26.04 (30.40)	NA
Protective factors				
Social support (FACT‐G)	0–24	17.33 (3.77)		NA
Partner support (DCI‐SDCP)	5–25	17.49 (4.10)	19.74 (3.09)	< 0.001
Continuity of care (CQI)	0–100	73.51 (23.89)	77.88 (23.41)	< 0.001

Abbreviations: CQI = Consumer Quality index (partner 43 missings; Patient 29 missings); DCI‐SDCP = Supportive Dyadic Coping of Partner subscale of Dyadic coping inventory (partner 33 missings; Patient 44 missings); EORTC QLQ‐C30 = European organization for research and treatment of cancer quality of life questionnaire core 30 items (partner 19 missings; Patient 6 missings); FACT‐G = Functional assessment of cancer therapy general (partner 18 missings); ZARIT‐12 = ZARIT burden scale (partner 26 missings).

**Caregiver burden** was measured using the Zarit Burden interview, 12‐item version (ZARIT‐12) [23]. This scale assesses the perceived impact of caregiving on emotional, physical, and social well‐being. Items are rated on a five‐point Likert scale ranging from 0 (“Never”) to 4 (“Nearly always”), with higher scores indicating greater burden.

**Emotional, physical** and **sleep problems** were assessed using subscales from the European Organization for Research and Treatment of Cancer Quality of Life Questionnaire Core 30 (EORTC QLQ‐C30) [24]. The four‐item Emotional functioning (e.g., “Did you worry?”), five‐item physical functioning (e.g., “Do you have any trouble taking a short walk outside of the house?”) and one‐item sleep problems subscales (1 item: “Have you had trouble sleeping?” are scored on a four‐point Likert scale (1 = “Not at all” to 4 = “Very much”) and linearly transformed to a 0–100 scale. Note that we deviate from the QLQ‐C30 scoring manual. Because the emotional and physical functioning items capture problems rather than positive functioning, we did not reverse code these scales to reflect better functioning. Instead, higher scores indicate greater emotional and physical problems, enabling us to conceptualize it as risk factors (instead of protective factors) of caregiver burden.

**Social** **support** was measured using the Functional Assessment of Cancer Therapy–General (FACT‐G) [25], which includes a seven‐item social/family well‐being subscale. Responses are given on a five‐point Likert scale (0 = “Not at all” to 4 = “Very much”), with higher scores indicating more perceived social support.

**Partner** **support** was assessed using the Supportive Dyadic Coping of the Partner subscale from the Dyadic Coping Inventory (DCI‐SDCP) [26]. This five‐item subscale evaluates how much emotional and instrumental support a person perceives from their partner during stress. Items are rated on a five‐point Likert scale (1 = “Very rarely” to 5 = “Very often”), with higher scores indicating greater perceived partner support.

**Continuity of care** was measured using selected items from the Consumer Quality Index (CQI) [27]. This Dutch instrument evaluates patients' experiences with healthcare delivery. The item relevant to continuity (i.e., “does the care of the different healthcare providers you are in contact with align?”) was rated on a four‐point Likert scale and linearly transformed to a 0–100 scale, where higher scores reflect better perceived continuity of care.

In the present sample, internal consistency was good for the Zarit Burden Interview‐12 (Cronbach ’s *α* = 0.87), the EORTC QLQ‐C30 emotional problems subscale (patients' *α* = 0.86 and partners' *α* = 0.84) and physical problems subscale (*α* = 0.83), the FACT‐G social support subscale (*α* = 0.81), and the DCI partner support subscale (patients' *α* = 0.79 and partners' *α* = 0.87). Reliability indices are not available for single‐item measures (e.g., sleep problems, continuity of care) because internal consistency cannot be calculated for single‐item indicators.

### 2.6 Data Analysis

Data were analyzed using *R*, version 4.5.0 on August 11, 2025. Descriptive statistics were used to summarize participant characteristics and study variables (means and standard deviations for continuous variables; frequencies and percentages for categorical variables). Given the ordinal response formats of the questionnaires and the non‐normal distribution of several variables, data were treated as ordinal and Spearman rank correlations were used for network estimation, in line with methodological recommendations for psychological network analysis.

We estimated a partial correlation network or so‐called Gaussian Graphical Model (GGM) to the data. In the network, “nodes” represent the selected variables while “edges” represent the connections between nodes. These connections are based on a partial correlation between two nodes, after conditioning on (i.e., controlling for) all the other variables in the network. As recommended by Epskamp and Fried [18], we accounted for the ordinal, nonnormal nature of the data by using Spearman rank correlations when estimating the network structure. Among participants who filled out the baseline questionnaire, 4.1% of responses were missing across all variables. The use of (multiple) imputation has not yet been studied in detail in network models [19]. Assuming that responses are missing at random, we used pairwise complete observations to handle missing data.

For network estimation, we used the estimateNetwork function with EBICglasso as an estimator in the bootnet package (version 1.6) [28]. To control for potential spurious associations, the estimation procedure uses the graphical LASSO regularization method based on the Extended Bayesian Information Criterion. Networks have been visualized using the qgraph package (version 1.9.8) [29]. To facilitate the interpretability of network visualization, we used different colors to highlight the categorization of nodes, and curved the edges that would otherwise cross nodes. The layout used was the automatically generated layout based on the Fruchterman–Reingold algorithm [30]. No specific minimum, maximum, or cut values have been used for network visualization.

We also explored whether adding covariates of sex and age to the network model would result in a similar network structure. Previous research has shown that female sex and younger age predict higher levels of caregiver burden [15, 31]. Due to the inclusion of a categorical variable, a mixed graphical model was estimated including these covariates (version 1.6) [28].

### 2.7 Network Stability, Node Centrality, and Edge Weights

As recommended by Borsboom and colleagues [32], we investigated the robustness and replicability of results by performing accuracy and stability checks using the *R* package bootnet [28]. To further quantify how well a node is directly connected to other nodes, we investigated strength as a centrality measure [33, 34]. Strength centrality refers to how strongly a node is directly connected to other nodes in the network by summing all absolute edge weights of edges connected to the given node. We assessed the accuracy of the strength centrality estimates by using case‐drop bootstrapping based on 1000 bootstrap samples [28]. To ensure interpretable differences in centrality and to assess whether certain edges were stronger and stood out in the network structure, we used the bootstrapped difference test.

### 2.8 Shortest Paths

Subsequently, we computed a network illustrating the shortest paths from caregiver burden toward all other nodes. In comparison to the first network, this network allows clear identification of the direct and indirect pathways between caregiver burden and its dyadic risk and protective factors. Path lengths are based on the inverse of the absolute edge weights raised to the power alpha, indicating that stronger edges reflect shorter paths [29]. The shortest path between two nodes is computed using Dijkstra's algorithm and represents the minimum number of steps needed to go as quickly as possible from one node to the other [35]. Given that the partial correlation between one node and all other nodes can be interpreted as predictive effects, any connection between two nodes that are linked by another node (e.g., the link between A and B in the pathway A–X–B) can be interpreted as an indirect effect between the two nodes.

## 3 Results

### 3.1 Study Sample

Of the 749 patient–relative pairs in the eQuiPe study, we included 564 (75.3%) patient–partner couples (see Supplementary Material 1 for a description of difference between the included patient‐partner couples and excluded patient‐relative pairs). The 185 excluded pairs were mostly patients' children (63.7%), other relatives (21.8%), or friends (12.9%). Partner caregivers had a mean age of 64.52 years (SD = 9.56) and were mostly female (54.6%). Patients averaged 65.17 years (SD = 9.14), with 44.15% female. See Table 2 for sociodemographic and clinical characteristics. Patients were most often diagnosed with lung (*n* = 154, 27.3%), colorectal (*n* = 104, 18.4%), breast (*n* = 73, 12.9%), or prostate cancer (*n* = 76, 13.5%). Partner caregivers reported significantly higher emotional problems, and lower continuity of care and partner support than patients (See Table 1).

**TABLE 2:** Baseline sociodemographic and clinical characteristics of 564 partners and patients.

Characteristics	Partner caregiver *N* (%)	Patient *N* (%)
Age in years, M (SD)	64.52 (9.56)	65.17 (9.14)
Sex (female)	308 (54.60)	249 (44.15)
Educational level		
Low	160 (28.60)	160 (28.62)
Medium	253 (45.20)	227 (40.61)
High	147 (26.30)	172 (30.77)
Children living at home (yes)	87 (17.90)	80 (17.32)
Caregiving hours/week	29.31 (41.03)	31.59 (46.78)
Length of the relationship		
< 1 year	1 (0.2)	1 (0.2)
1–5 years	7 (1.2)	6 (1.1)
≥ 5 years	555 (98.6)	523 (92.7)
Relationship satisfaction (dyadic adjustment scale with 0–6 range), M (SD)	4.00 (1.15)	4.07 (1.12)
Cancer type		
Lung		154 (27.3)
Colorectal cancer		104 (18.4)
Breast		73 (12.9)
Prostate		76 (13.5)
Other		157 (27.8)
Time since diagnosis in months		43.55 (54.97)
Perceived prognosis by patient		
< 1 year		70 (13.57)
> 1 year		148 (28.68)
Not life threatening		18 (3.49)
Not communicated/I do not know		210 (40.70)
Not wanting to know		70 (13.57)
Treatment in the last 3 months		
Chemotherapy		343 (61.03)
Radiotherapy		81 (14.41)
Surgery		18 (3.20)
Immunotherapy		156 (27.76)
Other treatment		103 (18.36)
No treatment		34 (6.05)

*Note:* Missings: age (partner 18; patient), educational level (partner 4; Patient 5), children at home (partner 77; Patient 102); caregiving hours (partner 266; Patient 293); length relationship (partner 1; Patient 34), relationship satisfaction (partner 23; Patient 42), time since diagnosis (Patient 6), prognosis (Patient 48). Caregiving hours per week reflects partner caregivers reported hours of care provided per week and patients reported hours of care received per week.

### 3.2 Network Structure of Caregiver Burden

**FIGURE 1:**
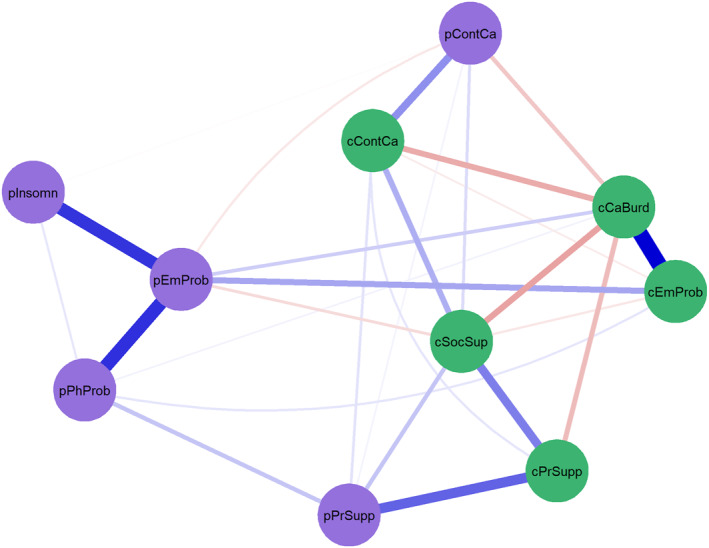
Network structure of dyadic risk and protective factors of caregiver burden (*n* = 564). The stronger the connection between two nodes, the thicker and more saturated the edge. Positive and negative connections are denoted by blue and red edges, respectively. Partner caregiver nodes are depicted in green and include cCaBurd = caregiver burden; cEmProb = emotional problems; cSocSup = social support; cPrSupp = partner support; cContCa = continuity of care. Patient nodes are depicted in purple and include pEmProb = emotional problems; pPhProb = physical problems; pInsomn = insomnia; pPrSupp = partner support; pContCa = continuity of care.

The network visualization is presented in Figure 1. In the nonparametric bootstrap analysis of edge weights (see Supporting Information S1) the intervals of the edges close to zero were quite wide, indicating that the estimation of the smaller edge weights should be interpreted with caution as they may differ from the obtained values. Supporting Information S1 presents the edge weights of the network. Of the total number of 45 possible edges in a fully connected network, 27 connections were significantly different from zero. This refers to a network density of 0.60 (27/45) with a mean edge weight of 0.037. Edge weights ranged from −0.133 (*caregiver burden – caregiver social* *support*) to 0.364 (*caregiver burden – caregiver emotional problems*).

### 3.3 Network Centrality and Edges

**FIGURE 2:**
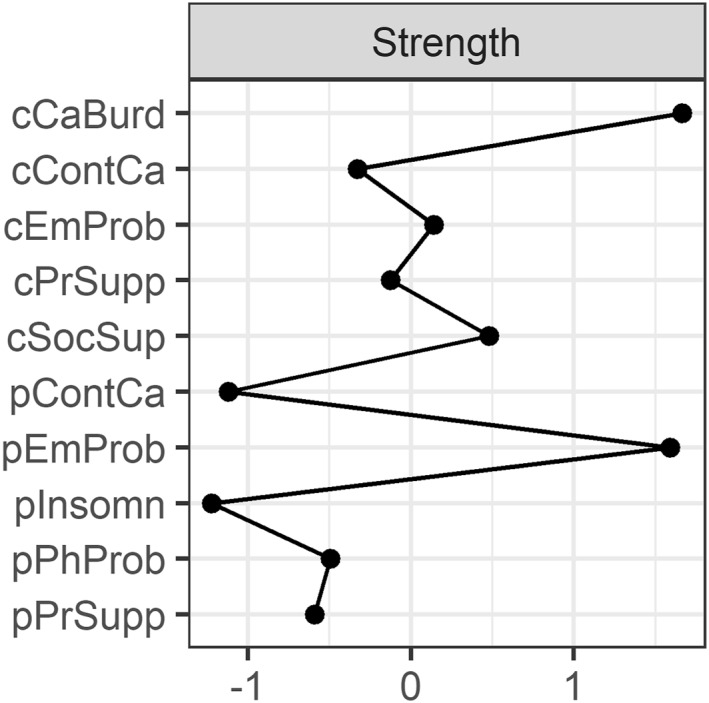
Strength centrality of each node in the network. Node strength refers to how strongly a node is directly connected to other nodes in the network (i.e., by summing all absolute edge weights of edges connected to the given node). All values are standardized, and higher values indicate greater centrality in the network. Partner caregiver nodes include cCaBurd = caregiver burden; cEmProb = emotional problems; cSocSup = social support; cPrSupp = partner support; cContCa = continuity of care. Patient nodes include pEmProb = emotional problems; pPhProb = physical problems; pInsomn = insomnia; pPrSupp = partner support; pContCa = continuity of care.

Strength centrality is presented in Figure 2. The bootstrapped difference test (see Supporting Information S1) indicated *caregiver burden* and *patient emotional problems* were more central than all other nodes. This means that *these nodes* were the most strongly connected nodes in the network with *caregiver burden* having seven edges varying between −0.113 and 0.364 and *patient emotional problems* having six edges varying between −0.053 and 0.297. The least central nodes were *patient insomnia* and *patient continuity of care.*

The bootstrapped difference test (see Supporting Information S1) identified five edges as significantly different from most other edges in the network. These included connections with and among risk factors: *caregiver burden – caregiver emotional problems* (0.364), *patient emotional problems – patient physical problems* (0.297), *patient emotional problems – patient insomnia* (0.291); and connections among protective factors: *caregiver partner* *support – patient partner* *support* (0.224), and *caregiver social* *support – caregiver partner* *support* (0.184). For example, this means that, considering all other nodes in the network, high levels of *caregiver burden* most strongly co‐occurred with high levels of *caregiver emotional problems*. Or put differently, partner caregivers who reported higher levels of caregiver burden tended to report higher levels of emotional problems.

After adding sex and age as covariates to the model, the network showed a similar structure. The same edges connected *caregiver burden* to the other nodes in the network (see Supporting Information S1). Partners' *sex* was not connected to any of the nodes in the network. Partners*' age* was connected to several risk and protective factors of the patient: *patient physical problems, patient partner* *support and patient continuity of care*. This means that older partner caregivers were more often partnered with a patient who experienced more physical problems, more partner support and more continuity of care. In addition, partner caregivers' *age* was related to *caregiver burden* and *partner emotional problems*: older partners were more likely to experience less caregiver burden and less emotional problems.

### 3.4 Shortest Paths of Caregiver Burden

**FIGURE 3:**
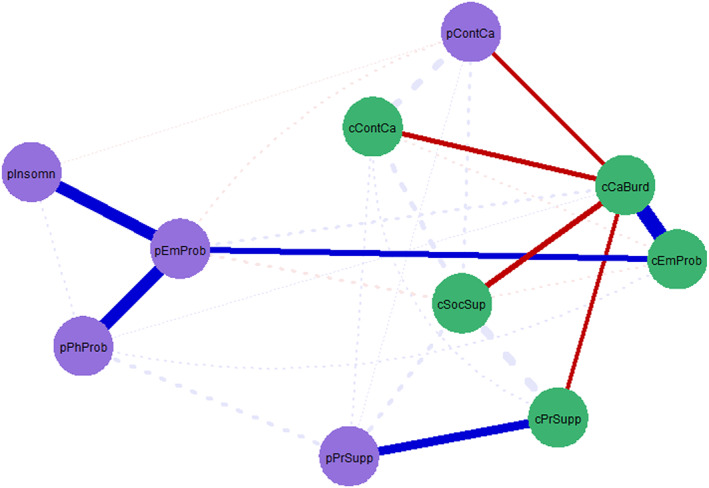
Shortest paths from caregiver burden to all other nodes. Shortest paths reflect the minimum number of steps to go as quickly as possible from caregiver burden to all other nodes. Shortest paths are highlighted. The stronger the connection between two nodes, the thicker the edge. Positive and negative connections are denoted by blue and red edges, respectively. Partner caregiver nodes are depicted in green and include cCaBurd = caregiver burden; cEmProb = emotional problems; cSocSup = social support; cPrSupp = partner support; cContCa = continuity of care. Patient nodes are depicted in purple and include pEmProb = emotional problems; pPhProb = physical problems; pInsomn = insomnia; pPrSupp = partner support; pContCa = continuity of care.

The results of the shortest path analysis are visualized in Figure 3, reflecting the minimum number of steps to go to as quickly as possible from *caregiver burden* to all other nodes. Findings indicated that *caregiver burden* is directly connected to all risk (*caregiver emotional problems*) and protective factors (*caregiver social* *support, caregiver partner* *support, caregiver continuity of care*) reported by partner caregivers. Regarding patient risk and protective factors, *caregiver burden* was directly connected to *patient continuity of care* and indirectly connected to *patient emotional problems* (via *caregiver emotional problems*), *patient insomnia* and *patient physical problems* (via *caregiver emotional problems* and *patient emotional problems*), and *patient partner* *support* (via *caregiver partner* *support*). Note that although there is a direct edge between *caregiver burden* and *patient physical problems*, the shortest path runs via *caregiver emotional problems* and *patient emotional problems* because the edges with *caregiver and patient emotional problems* (*caregiver burden* – *caregiver emotional problems* (0.364); *caregiver emotional problems* – *patient emotional problems* (0.127); *patient emotional problems – patient physical problems* (0.297)) are stronger than the direct edge between *caregiver burden* and *patient physical problems* (0.019). The relationship between *caregiver burden* and *patient physical problems* thus appears to be indirectly connected via *caregiver* and *patient emotional problems*.

## 4 Discussion

The present study applied network analysis on cross‐sectional data from 564 advanced cancer patients and their partner caregivers to explore the interconnectedness of dyadic risk and protective factors of caregiver burden. Partner caregivers reported more emotional problems, less continuity of care and less partner support than patients. By studying what dyadic risk and protective factors are directly and strongly connected to caregiver burden, we aimed to gain insight into which constructs could be intervened upon to support partners with caregiver burden. Shortest path analysis revealed that caregiver burden was mainly connected to caregiver‐related risk (i.e., emotional problems) and protective factors (i.e., continuity of care, social support, partner support). Continuity of care was the only protective factor of the patient that was directly connected to lower caregiver burden. Patients' physical and sleep problems were indirectly connected to partners' caregiver burden via emotional problems of the patient and partner.

Our findings revealed that continuity of care as perceived by the patient as well as by the partner were independently connected to caregiver burden, suggesting that when not only the patient but also the partner experiences more continuity of care, the partner experiences less caregiver burden. Previous research has underscored the importance of continuity of care for both patients as well as partner caregivers in palliative care, showing that both groups identified continuity of care as one of the most essential aspects of healthcare [14]. In addition, we found that partners perceived less continuity of care than patients. Better continuity of care may reduce caregiver burden by lowering uncertainty in both patient and partner, minimizing contradictory information, and reducing the need for partners to coordinate care between multiple healthcare providers. When care is perceived as well‐aligned, both patients and partner caregivers may feel more supported and less solely responsible for monitoring and managing the patient's condition, thereby reducing emotional strain. Together, these findings suggest that by involving partners more in palliative care, we could help lower their caregiver burden. Such involvement in consultations can increase confidence in their caregiving role and improve their ability to take care of the patient [36]. Such involvement does not appear to exacerbate caregiver burden but to alleviate it by fostering a sense of agency and inclusion in the care process [36].

None of the other patient risk and protective factors were directly connected to caregiver burden. Rather, shortest path analysis revealed that the association between patients' and partners' emotional problems connect the physical problems and sleep problems of the patient to caregiver burden of the partner. These results are in line with the literature showing how patients' and partners' distress levels are robustly correlated [9], indicating that when the patient experiences more emotional problems, the partner experiences more emotional problems. Interestingly, the indirect pathway in the present network model linking patient physical and sleep problems to partners' caregiver burden ‐running via both patient and partner emotional problems‐further underscores the view that couples function as an interdependent emotional system rather than as two isolated individuals. This implies that when we want to support partners in their caregiver burden, we need to target both the partner caregiver as well as the patient. A recent meta‐analysis found that dyadic interventions including communication and support components are effective in reducing the psychological distress of both members of the couple [37]. Another meta‐analysis found that among family caregivers of advanced lung cancer patients, dyadic interventions seem to also reduce caregiver burden [38].

### 4.1 Study Limitations

Study findings should be interpreted considering some limitations. Patients who felt that the burden on their relative(s) was already high may have refused to ask their relatives to participate. Therefore, our results may underestimate the caregiver burden of partners. Moreover, it is likely that patient and partners completed the questionnaires together, potentially influencing one another's responses. In addition, the strength of the statistical evidence for the presence or absence of a connection in cross‐sectional networks is strongly determined by the sample size relative to the number of possible connections (i.e., the relative sample size) of the network [39]. Our network model with 12.5 observations per possible connection (i.e., 564 couples/45 possible connections), is relatively small. Furthermore, while the eQuiPe study offered a rich dataset, we were unable to include other nodes that have been shown to contribute to caregiver burden, such as patient's illness acceptance or partner's physical problems [5, 12]. A further limitation concerns measurement heterogeneity. Patient sleep problems and continuity of care were assessed using single‐item measures, which typically have lower reliability and higher measurement error than multi‐item scales. Consequently, the relatively peripheral position of these nodes in the network may partly reflect measurement limitations rather than true clinical irrelevance. The findings should also be interpreted within the scope of partner caregiving. Caregiver burden among partners may differ from that experienced by adult children or other relatives, and the present results should not be generalized to all caregiver types. Moreover, the cross‐sectional, between‐subjects design employed in the current study precludes causal inference and limits the generalizability of findings to the individual level. To address these limitations, future research could adopt time‐series designs, such as ecological momentary assessment (EMA), to investigate the dynamic interplay of risk and protective factors associated with caregiver burden over time. Such designs could test, for example, whether emotional functioning at one time point predicts subsequent changes in caregiver burden.

In addition, it is important to acknowledge the methodological implications of using a network approach in this context. Importantly, the network and shortest‐path approaches offer insights that would be difficult to obtain using traditional regression‐based methods. Whereas conventional analyses typically examine independent predictors of caregiver burden, the network approach conceptualizes caregiver burden as embedded within a system of mutually interacting factors. For example, patient physical and sleep problems were not directly connected to caregiver burden but operated indirectly through emotional problems of both partners. Such indirect pathways are likely to remain obscured in traditional models focusing solely on direct effects.

### 4.2 Clinical Implications

Although the present study is based on cross‐sectional baseline data and therefore does not allow causal inference or conclusions about change over time, the identified network structure highlights potentially relevant points of intervention at the group level. The clinical implications outlined below should thus be interpreted as hypotheses for intervention targets rather than as evidence of causal effects, warranting further evaluation in longitudinal and intervention studies. Improving continuity of care appeared as a key target for intervention. This remains a complex endeavor, particularly in the context of advanced cancer, where care trajectories are often complicated by psychological and physical comorbidities, polypharmacy, and the involvement of multiple healthcare providers from different care organizations. Additionally, workforce shortages and high clinical workloads may hinder effective interprofessional collaboration across hospital departments and external care organizations. Interventions aimed at improving continuity of care may include the use of dedicated care coordinators or case managers, structured communication between healthcare providers, and the meaningful involvement of partner caregivers in clinical consultations [40, 41]. Partners are well‐positioned to provide nuanced insights into the patient's condition and care needs within the home environment during such consultations, thereby facilitating more tailored, coordinated and continued care planning, which in turn can reduce partners' caregiver burden.

Our findings also highlight the importance of addressing caregiver burden within the context of the couple's shared emotional functioning. Interventions aimed solely at the partner caregiver may overlook the relational context in which caregiver burden develops. Instead, dyadic approaches that simultaneously target patient and partner emotional functioning are warranted. Dyadic interventions may, for example, include joint counseling sessions, communication training, or dyadic coping interventions delivered face‐to‐face or via blended formats. Evidence from recent meta‐analyses demonstrates that dyadic interventions can effectively reduce psychological distress in both members of the couple and alleviate caregiver burden [37, 38].

## 5 Conclusion

In conclusion, this network study highlights continuity of care and the interdependence between patient and partner emotional problems as key dyadic protective and risk factors of caregiver burden. Patient physical and sleep problems were linked to caregiver burden primarily through emotional pathways. Future research should employ intensive longitudinal designs, such as ecological momentary assessment, to examine how these dyadic processes unfold over time and to inform timely, targeted interventions.

## Conflicts of Interest

The authors declare no conflicts of interest.

## Supporting information


Supporting Information S1

## Data Availability

The data that support the findings of this study are available on request from the corresponding author. The data are not publicly available due to privacy or ethical restrictions.
