# Supplementary material for: Dyadic Risk and Protective Factors of Caregiver Burden Among Partners of Patients With Advanced Cancer: A Network Approach
Source: Psychooncology. 2026 Feb 5;35(2):e70403. doi: 10.1002/pon.70403 (PMC12876055; doi:10.1002/pon.70403)
Supplement: Supplementary file 1 — Supporting Information S1 [file PON-35-e70403-s001.docx]

**Supplementary Material 1. Description of how included patient-partner couples differ from patient-other relative couples.**

Of the 749 patient–relative pairs in the eQuiPe study, we included 564 (75.3%) patient–partner couples (see Table 2 for sociodemographic and clinical characteristics). The 185 excluded pairs were mostly patients’ children (63.7%), other relatives (21.8%), or friends (12.9%). Included partner caregivers (n=564) were more often male (n=315 (55.85%) versus n=59 (33.96%), p<0.001), were older (M=64.52 (SD=9.56) versus M=47.53 (SD=14.47), p<0.001), had less often children living at home (n=87 (17.86%) versus n=76 (64.41%), p<0.001), were more often lower educated (n=160 ( 28.57%) versus n=33 (18.54%), p=0.005), and perceived higher continuity of care (M=73.51 (SD=23.89) versus Mean=67.47 (SD=23.20), p=0.004) compared to excluded family caregivers (n=185). There were no differences among the other network variables (Table 1) and baseline characteristics (Table 2). Included patients (those with a partner caregiver) were more often male (n=315 (55.85%) versus n =59 (32.96%), p<0.001), and were less often diagnosed with lung and breast, and more often with prostate cancer (p = 0.01) than excluded patients (those with a family caregiver). There were no additional differences among the other network variables and baseline characteristics.

**Supplementary Table 1. Edge weights.**

|  | cCaBurd | cEmProb | cSocSup | cPrSupp | cContCa | pEmProb | pPhProb | pInsomn | pPrSupp | pContCa |
| --- | --- | --- | --- | --- | --- | --- | --- | --- | --- | --- |
| cCaBurd | 0.000 | 0.364 | -0.133 | -0.097 | -0.121 | 0.071 | 0.019 | 0.000 | 0.000 | -0.082 |
| cEmProb | 0.364 | 0.000 | -0.036 | 0.000 | -0.029 | 0.127 | 0.038 | 0.000 | 0.000 | 0.000 |
| cSocSup | -0.133 | -0.036 | 0.000 | 0.185 | 0.115 | -0.053 | 0.000 | 0.000 | 0.085 | 0.051 |
| cPrSupp | -0.097 | 0.000 | 0.185 | 0.000 | 0.037 | 0.000 | 0.000 | 0.000 | 0.224 | 0.000 |
| cContCa | 0.120 | -0.029 | 0.115 | 0.037 | 0.000 | 0.000 | 0.000 | 0.000 | 0.042 | 0.160 |
| pEmProb | 0.071 | 0.127 | -0.053 | 0.000 | 0.000 | 0.000 | 0.297 | 0.291 | 0.000 | -0.034 |
| pPhProb | 0.019 | 0.038 | 0.000 | 0.000 | 0.000 | 0.297 | 0.000 | 0.034 | 0.083 | 0.000 |
| pInsomn | 0.000 | 0.000 | 0.000 | 0.000 | 0.000 | 0.291 | 0.034 | 0.000 | 0.000 | -0.006 |
| pPrSupp | 0.000 | 0.000 | 0.085 | 0.224 | 0.042 | 0.000 | 0.083 | 0.000 | 0.000 | 0.018 |
| pContCa | -0.082 | 0.000 | 0.051 | 0.000 | 0.160 | -0.034 | 0.000 | -0.006 | 0.018 | 0.000 |

Note. cCaBurd = caregiver burden; cEmProb = emotional problems; cSocSup = social support; cPrSupp = partner support; cContCa = continuity of care. Patient nodes are depicted in purple and include pEmProb = emotional problems; pPhProb = physical problems; pInsomn = insomnia; pPrSupp = partner support; pContCa = continuity of care.

**Supplementary Figure 1. The 95% CI of edge weights.**


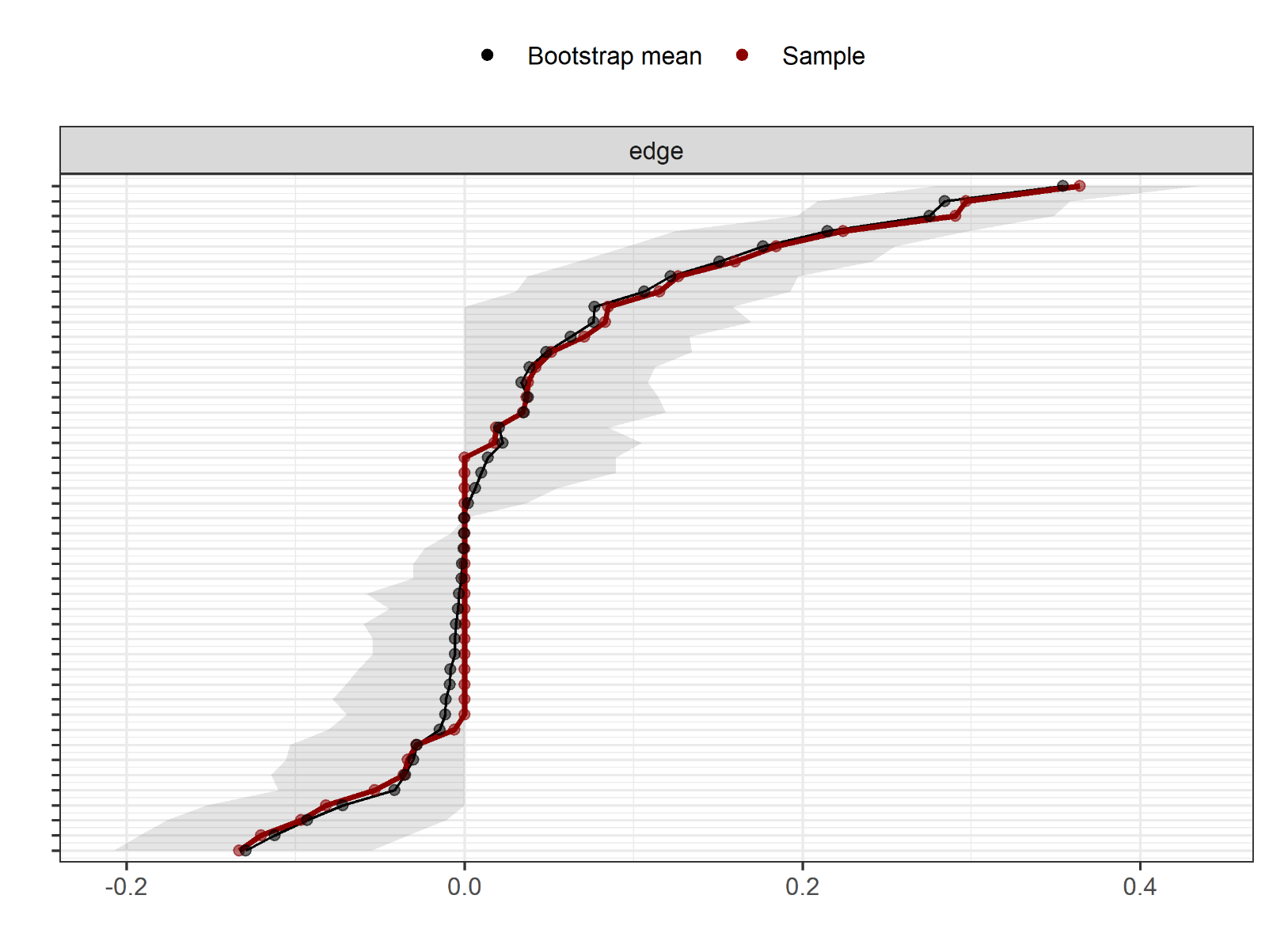


*Note.* The intervals of the edges close to zero were quite wide, indicating that the estimation of the smaller edge weights should be interpreted with caution as they may differ from the obtained values.

**Supplementary Figure 2. Significant differences between strength centrality of nodes.**


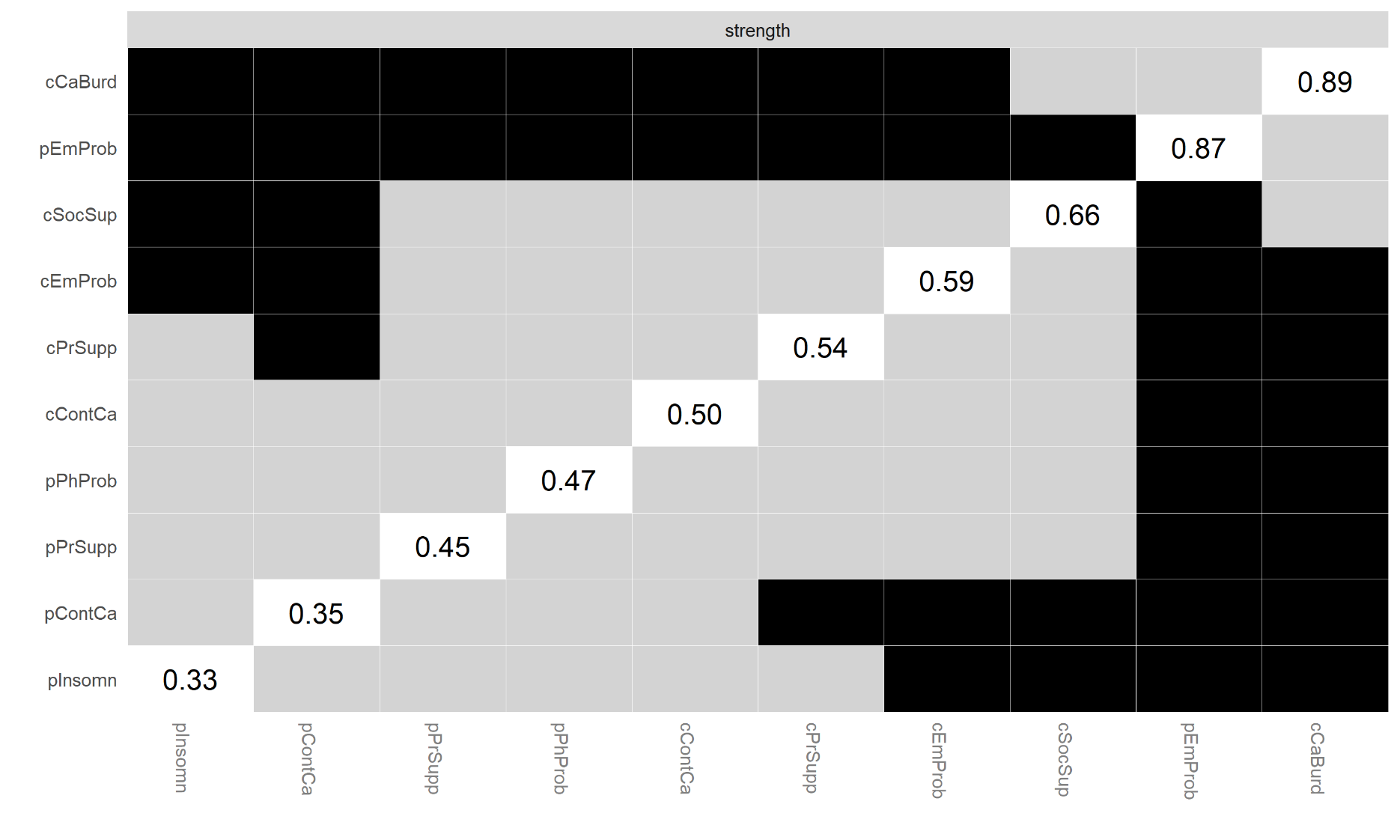


*Note.* A black square depicts a significant difference between two edges. A grey square depicts a non-significant difference between two edges.

**Supplementary Figure 3. Significant differences between edge weights.**


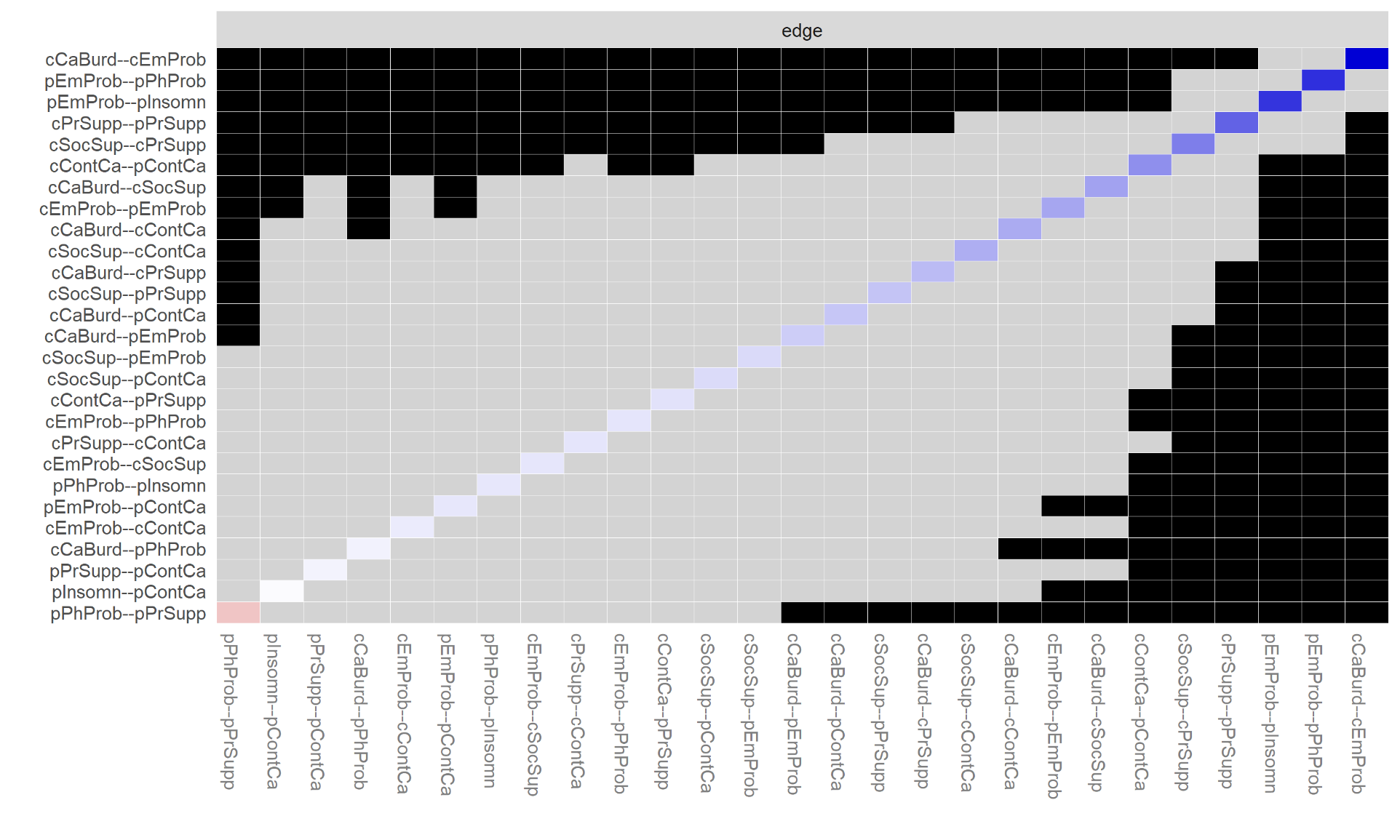


*Note.* A black square depicts a significant difference between two edges. A grey square depicts a non-significant difference between two edges.

**Supplementary Figure 4. Network model of FCR components including caregiver age and sex as covariates.**

*
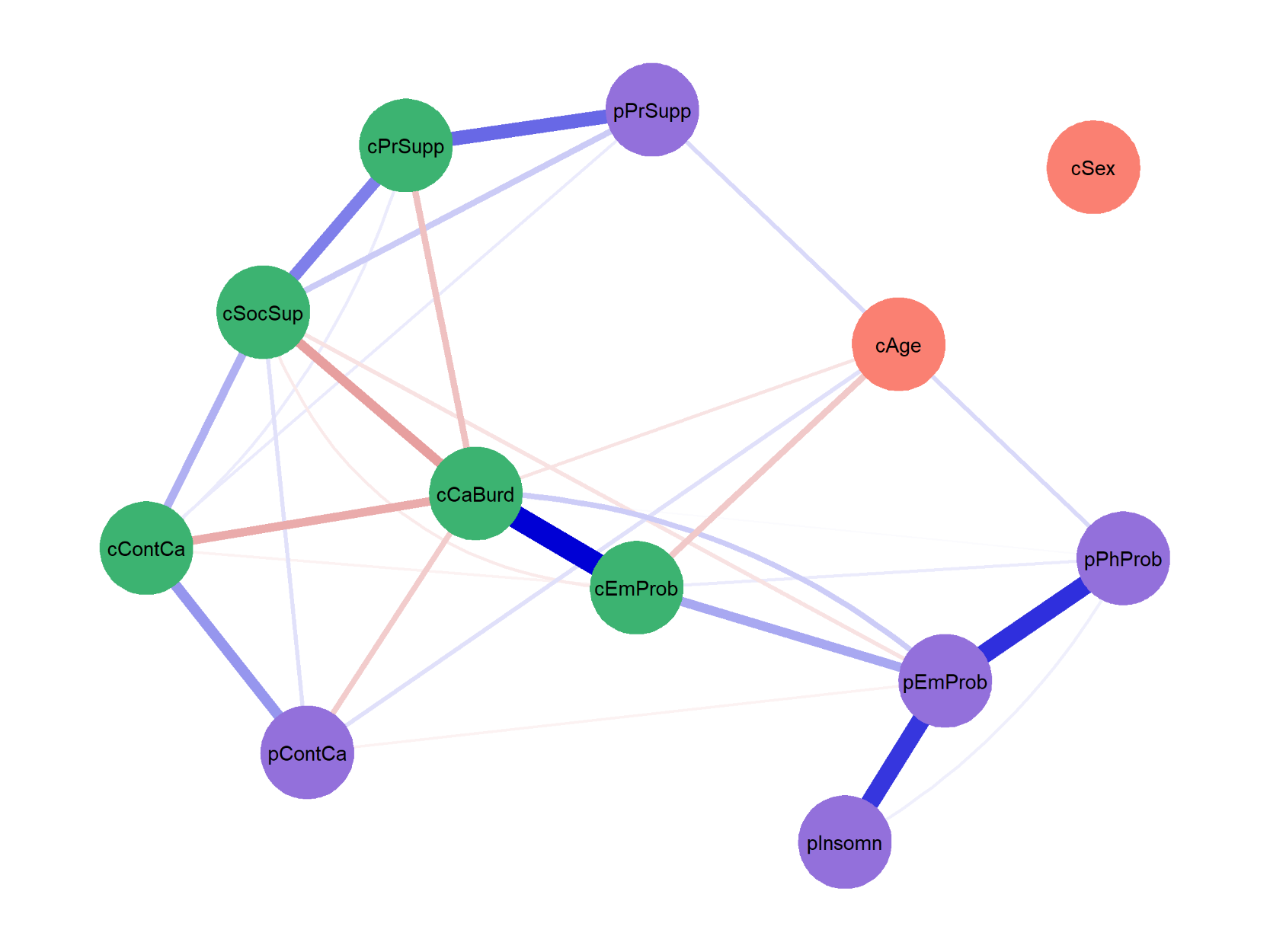
Note.* The depicted network is a mixed graphical model in order to include a categorical variable (i.e. sex). This estimation method handles missing values via listwise rather than pairwise deletion. Therefore, the sample size in this network (n=419) is smaller than the Gaussian Graphical Model (n=564) as depicted in Figure 1 of the manuscript.
